# Evaluating the Feasibility of Measuring Travel to School Using a Wearable Camera

**DOI:** 10.1016/j.amepre.2012.07.027

**Published:** 2012-11

**Authors:** Paul Kelly, Aiden R. Doherty, Alex Hamilton, Anne Matthews, Alan M. Batterham, Michael Nelson, Charlie Foster, Gill Cowburn

**Affiliations:** a British Heart Foundation Health Promotion Research Group, University of Oxford, United Kingdom; b Health and Social Care Institute, Teesside University, United Kingdom; c The School Food Trust, United Kingdom

## Abstract

**Background:**

The school journey is often studied in relation to health outcomes in children and adolescents. Self-report is the most common measurement tool.

**Purpose:**

To investigate the error on self-reported journey duration in adolescents, using a wearable digital camera (Microsoft SenseCam).

**Methods:**

During March–May 2011, participants (*n*=17; aged 13–15 years) from four schools wore wearable cameras to and from school for 1 week. The device automatically records time-stamped, first-person point-of-view images, without any action from the wearer. Participants also completed a researcher-administered self-report travel survey over the same period. Analysis took place in November 2011. Within- and between-subjects correlation coefficients and Bland-Altman 95% limits of agreement were derived, accounting for the multiple observations per individual.

**Results:**

Self-report data were collected for 150 journey stages and SenseCam data for 135 (90%) of these. The within-subjects correlation coefficient for journey duration was 0.89 (95% CI=0.84, 0.93). The between-subjects correlation coefficient was 0.92 (95% CI=0.79, 0.97). The mean difference (bias) between methods at the whole sample level was small (10 seconds per journey, 95% CI= −33, 53). The wide limits of agreement (±501 seconds, 95% CI= −491, 511) reveal large random error.

**Conclusions:**

Compared to direct observation from images, self-reported journey duration is accurate at the mean group level but imprecise at the level of the individual participant.

## Background

Physical activity is associated with important health outcomes in children, including body composition, type 2 diabetes, and cardiovascular fitness.^1–6^ Active travel to school, including walking and cycling, can be an important contributor to physical activity levels.^7,8^ Conversely, travel in motor vehicles is a sedentary behavior representing a lost opportunity for physical activity.^9^

Research into school-related travel behavior faces methodologic challenges and valid, accurate measures are required.^10,11^ Self-report is the most common tool, but its accuracy and precision are debated.^10,12–15^ Better understanding of the accuracy and potential error in self-report will improve the calculation of health associations and the ability to detect changes in behavior.

**Figure 1:**
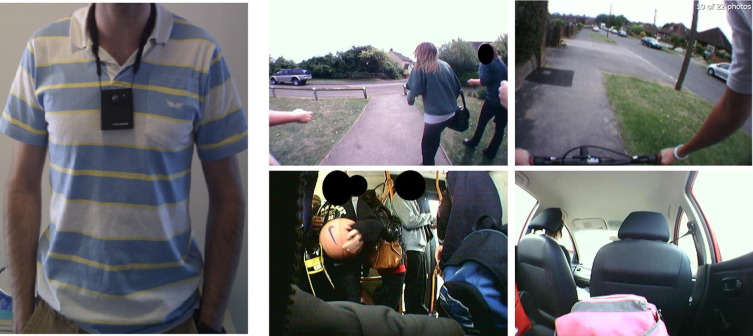
The Microsoft SenseCam digital camera with a sample of school-journey images *Note:* Images shown were collected in the current study. Left: SenseCam (this wearable device weighs 175 g and passively captures approximately 3600 first-person point-of-view digital images per typical day). Clockwise from top center to bottom center: Images from the camera with the wearer walking, cycling, riding in a car, and riding in a bus. These travel images demonstrate the direct observation of school journey mode possible from the first-person point-of-view.

Wearable digital cameras are novel devices that may help develop such an understanding. Microsoft's SenseCam is one such camera, worn on a lanyard around the neck that automatically records time-stamped, first-person point-of-view images (Figure 1), without any action required by the wearer.^12^ It has been shown that a wearable camera can be used to estimate the bias and error on self-reported journey duration in adults.^16^ The present study aims to see if the protocol can be repeated in a younger population. This study has two research questions: (1) Can a wearable camera be used to measure travel behavior in a sample of teenagers aged 13–15 years? (2) What do wearable camera–recorded journey durations reveal about self-reported journey durations?

## Methods

### Participants

Volunteer participants (aged 13–15 years) were recruited from four secondary schools in England (Oxfordshire [three] and Yorkshire [one]) with a range of geographic locations (one city, two suburban, and one rural).

### Wearable Camera Protocol

Participants were asked to wear a camera to and from school for 1 week. They were provided with instructions on the practical issues with wearing the camera (e.g., wearing overly bulky clothing, weather, and securing the camera). Images were downloaded by researchers each morning and participants were given the option to delete any images they wished. Images were viewed using standard SenseCam software.^17^

Device reactivity can be an issue in physical activity assessment.^18^ To minimize this, participants were asked to wear the device throughout the school day so that they would become acclimated to the novelty. Participants were told that they could remove the device at any time or if asked to do so by a teacher, and could stop image recording for a 7-minute period by using a privacy button. Schools were informed fully about the project and written parental consent was obtained in advance. The device was configured to scramble images, which could then be unscrambled only on the laptop computers of the researchers administering this study.

### Travel Questionnaire Protocol

Participants completed a daily researcher-administered travel questionnaire on journey mode and duration. The questionnaire was a modified, unvalidated version of the National Travel Survey (NTS), a continuous annual United Kingdom (UK) survey.^11^ Participants were asked to include only travel time, and not other activities such as waiting for public transport. For this proof-of-concept study, a journey was defined as any transportation between any two locations of >3 minutes.

### Data Analysis

Data analysis took place in November 2011. From the images, each journey was manually identified and coded; the browser then automatically calculated the duration. The start image was identified visually by the researcher as the first image displaying travel (e.g., leaving the house or school or entering a vehicle); the end image was identified visually in the same way (e.g., arrival at front door or exiting vehicle). The full protocol is available on request.

A journey between school and home could contain multiple journey stages because of mode transition (e.g., walk; bus; walk) or journey-breaking (e.g., stopping at a friend's house or a shop). For this reason, the journey stage was the unit of analysis. For analysis, a journey stage was defined as purposeful movement lasting >3 minutes. If travel ceased for >3 minutes (e.g., stop in shop), the journey was considered broken.

Journey stages were coded independently by two researchers. An inter-rater reliability analysis using the Kappa statistic was performed to determine consistency among raters for mode, and using intraclass correlation coefficient (ICC; two-way random; absolute agreement) for duration. Bias between methods (group-level accuracy) was assessed using the paired *t*-statistic providing the mean difference between methods and its 95% CI. Individual journey–level agreement between methods was examined using Bland-Altman 95% limits of agreement,^19^ accounting for the clustered observations (journey stages) within participants. The correlation coefficient was calculated for the relationship between the journey durations estimated from the two methods using both within-subject^20^ and between-subject methods.^21^ All statistical analyses were conducted using *PASW* Statistics 18.0 and Medcalc 12.1.3 software packages.

### Ethics Approval

The present study received ethics approval from the Social Sciences and Humanities Inter-divisional Research Ethics Committee (IDREC) in accordance with the procedures laid down by the University of Oxford for ethical approval of all research involving human participants (IDREC reference number: SSD/CUREC1A/10-092).

## Results

Participants were volunteers aged 13–15 years (*n*=17; 11 girls, 6 boys). Data collection took place between March and May 2011.

### Question 1

Can digital image capture be used to measure travel behavior in a small sample of teenagers aged 13–15 years?

Figure 1 shows a sample of travel images collected. In all, 150 journey stages were reported in the travel survey. Wearable camera–recorded data for 135 (90%) of these journey stages (the 15 lost were due to camera not worn [*n*=8] or the lens was obscured for the start or end of the journey [*n*=7]). The wearable camera also recorded 12 journey stages that were missed by self-report. Table 1 shows the summary of journey stages and modes.

The inter-rater reliability for the raters for journey mode (categoric) assessed by image was found to be perfect agreement (Kappa=1.00). The inter-rater reliability for duration (continuous) assessed by image was also very high (intraclass correlation coefficient=0.989, 95% CI=0.985, 0.992), suggesting very good agreement. Data from individual interviews (not presented here) showed that participants reported few concerns with wearing and managing the camera. Although most participants reported feeling “self-conscious” on initial wearing, all agreed that they became comfortable and familiar with the equipment within a few hours.

### Question 2

What do wearable camera–recorded journey durations reveal about self-reported journey durations?

From the 135 journey stages, 31.4 hours of travel were reported (average stage=838 seconds; 13 minutes 58 seconds). The wearable camera recorded 31.1 hours (average stage=828 seconds; 13 minutes 48 seconds) from approximately 12,000 images of travel. At the group mean level, self-reported journey stage durations were 10 seconds longer per journey (95% CI= −33, 53; 95% limits of agreement= ±501 seconds, 95% CI= −491, 511).

**Figure 2:**
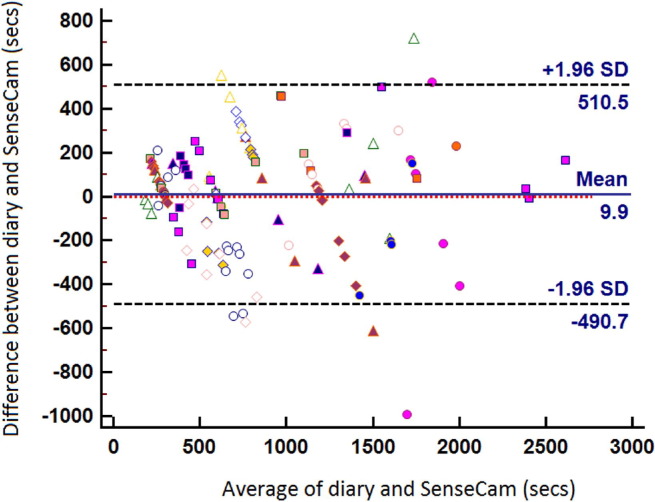
Limits-of-agreement (Bland-Altman) plot for self-reported journey duration and for journey duration recorded by wearable camera *Note:* There is one marker for each observation pair. Each point above the *y*=0 line indicates a journey stage that was over-reported in the diary, and each point below the line indicates a journey stage that was under-reported in comparison to wearable camera–recorded journey duration. The plot shows the small bias and wide limits of agreement. secs, seconds

Both the within-subject and the between-subject correlation between methods was strong (*r*=0.89, 95% CI=0.84, 0.93; and 0.92, 95% CI=0.79, 0.97, respectively). In physical activity measurement, a value >0.80 is said to demonstrate acceptable validity.^22^ A Bland-Altman plot of the between-method differences against the mean journey duration for the two methods for the whole sample (Figure 2) illustrates the small fixed bias of 10 seconds (over-reporting of journey duration independent of journey length) as revealed by the paired *t*-statistic analysis. Of the 135 journey stages, 79 (59%) were over-reported and appear above the *y*=0 line, whereas 56 (41%) were under-reported.

## Discussion

The current study demonstrates for the first time that a wearable camera is a feasible technique for use in a school travel setting, for multiple days of data collection. The obtained images give an objective assessment of travel mode and an accurate and reliable measure of duration. This study also demonstrated some issues associated with the device: there are particular settings with which participants are not comfortable wearing the camera (notably at a friend's house visited during the journey home); journeys cannot always be determined when light levels are very low; images can be lost when the lens is obscured by clothing; the device can be forgotten for some journeys; and the 10-second epoch between image capture introduces a small error on calculation of duration. Protocols should be developed to address these issues and minimize data loss.

The present study also explored the feasibility of processing and coding large amounts of image data manually. Using the SenseCam software, a trained researcher could code a single participant's data in approximately 30 minutes. For such techniques to be considered feasible in larger studies, automated or semi-automated data recognition systems will be required.

In addition, the current study aimed to compare self-reported journey duration and wearable camera–recorded journey duration. The comparison of mean difference showed that at the level of the study population, there is good agreement between the measures. The overall limits-of-agreement analysis (Figure 2) suggests that at a group level across all modes, there is very little bias on reporting.

This finding of little bias is in contrast with results from a previous study by the same researchers that showed journey durations to be over-reported by adults.^16^ It also is in contrast to studies^23–25^ showing self-reported journey durations as greater than those measured by GPS. A possible explanation is the difference in self-report protocol of a diary versus a researcher-led questionnaire. Alternatively, it may be that perceptions of journey duration differ in adult and school-aged populations.

The wide limits of agreement indicate a lack of precision for self-reported journey duration. This suggests that self-report is a poor measure of individual journey behavior, and that the two methods should not be used interchangeably. This random error at an individual journey level is thought to exist because the way in which journey time is remembered and then reported varies from person to person, from day to day, and from journey to journey.^26^

### Implications

The small difference in means between self-reported and wearable camera–recorded journey stage duration suggests that self-report may be a better measure of group journey behavior than previously thought. However, the wide limits of agreement (16 minutes 42 seconds on an average duration of 13 minutes 58 seconds) demonstrate large random errors at an individual level and suggest that self-report may be inappropriate for assessing individual journeys. Large random errors introduce noise and reduce sensitivity on any data, which has implications for comparing groups or individuals by time engaged in walking, cycling, or otherwise traveling to school. The successful demonstration of this novel method over multiple days of study opens the way for future investigation into other important health behaviors (e.g., sedentary or nutrition) in this age group.

### Strengths and Limitations

With just 135 journey stages from 17 volunteer participants, this is a proof-of-concept study and the results cannot be considered representative. Whether wearing this type of camera influences travel or reporting behavior requires investigation. The strength of the device is that journey mode can be assessed objectively with the mode and duration analysis showing extremely high agreement among raters. The device is unlikely to be suitable for large-scale studies and probably will remain a validation and improvement tool for existing self-report techniques. Focusing solely on active travel to school limits the present study; it has been demonstrated that on its own, active travel to school is unlikely to be a cost-effective strategy to combat health outcomes such as obesity.^27^ Future studies should look to address other domains of childhood physical activity.

### Future Study

Having demonstrated the feasibility of wearable cameras in research on active travel to school, the next step is to assess the accuracy and precision of self-reported journey behavior in a sufficiently powered and representative sample. Using 17 participants, this proof-of-concept study indicates that self-report may be a good population measure and a very poor individual measure. However, the results cannot be considered representative. As this type of wearable camera is currently unsuitable for large-scale studies (because of data processing and coding time) it should be considered as a validation tool for techniques that can be used on a large scale (such as self-report).

Therefore, a reasonable next step is to test the findings of the current study in a second, sufficiently powered study. From the SD of the differences between measures, it is estimated that approximately 100 participants wearing the device for 1 week will give enough measurements for the results to have a high level of confidence.^19^ Non-school-based travel also should be incorporated into future studies.

### Conclusion

A wearable camera can be used to investigate journey mode and duration for 1 week of travel to and from school in volunteers aged 13–15 years. Compared to direct observation of travel behavior from time-stamped images, self-reported journey duration is accurate at the mean group level but imprecise at the level of the individual participant.

## Figures and Tables

**Table 1:** Travel mode, frequency, self-reported duration, and SenseCam-recorded duration for journey stages (*n*=135) for both measures

Travel mode	Frequency	Average self-reported duration^a^	Average SenseCam-recorded duration^a^
Walking	79	886 (14:46)	843 (14:03)
Cycling	6	800 (13:20)	514 (08:34)
Car	27	484 (08:04)	495 (08:15)
Bus	23	1250 (20:50)	1098 (18:18)
Total	135	838 (13:58)	828 (13:48)

a Values are in seconds (minutes and seconds).
